# Family Interactions of Adolescents During the COVID‐19 Pandemic: Experiences in the Home Context

**DOI:** 10.1155/nrp/4886656

**Published:** 2026-03-03

**Authors:** Nayara Gonçalves Barbosa, Thaianne Cristine Gadagnoto, Lise Maria Carvalho Mendes, Tatiane Geralda André, Giovanna Cristina Machado Kayzuka, Diene Monique Carlos, Flávia Azevedo Gomes-Sponholz, Lucila Castanheira Nascimento

**Affiliations:** ^1^ College of Nursing, University of São Paulo, São Paulo, São Paulo, Brazil, usp.br; ^2^ College of Nursing, University of São Paulo at Ribeirão Preto, Ribeirão Preto, São Paulo, Brazil; ^3^ Public Health Nursing Post-Graduate Program, College of Nursing, University of São Paulo at Ribeirão Preto, Ribeirão Preto, São Paulo, Brazil

**Keywords:** adolescents, COVID-19, family, family nursing

## Abstract

**Objective:**

This study aims to analyze adolescents’ family interaction experiences and social dynamics in the context of social distancing measures during the COVID‐19 pandemic.

**Methods:**

This study uses a qualitative methodological approach based on symbolic interactionism, with convenience sampling. It involved students aged 10 to 19 from two public schools, using semistructured remote interviews analyzed through inductive thematic analysis.

**Results:**

The study included 22 adolescents, predominantly female. The themes were as follows: “Caught by surprise”: break with the world and the (re)discovery of home; the new routine at home: conflicts and tensions; opportunities to reflect and be grateful: between fears, worries, and gratitude; learning through adversity: times of reconciliation and resignification. The adolescents who participated faced a significant routine disruption, spending more time at home, which reshaped family interactions. This adjustment phase involved coping with new household responsibilities and reduced privacy, sparking intrafamily conflicts necessitating dialogue and reconciliation efforts.

**Conclusion:**

The COVID‐19 pandemic reshaped the family interactions and daily routines of adolescents, requiring them to adopt new roles and coping strategies to navigate emotional, social, and financial challenges. Despite moments of conflict and loss, the experience also fostered personal growth, resilience, and a renewed appreciation for family support during adversity.

## 1. Introduction

The coronavirus pandemic (SARS‐CoV‐2) caused intense biopsychosocial transformations worldwide and across the entire global population [1, 2]. During this period, social distancing was adopted in many countries as one of the primary measures to control the disease [3, 4]. However, this had significant repercussions on the mental health of the population, especially in adolescence, one of the most complex stages in the life cycle [2, 5, 6].

Adolescence comprises a phase of intense biopsychosocial transformations involved in the transition from childhood to adulthood [7]. According to the World Health Organization [8], adolescence is defined as the age group between 10 and 19 years. This stage is characterized by independence from parents, concurrent with increased time with peers, who become the primary source of interaction and influence [1]. The experience of the COVID‐19 pandemic had significant repercussions with the interruption of in‐person school activities and the separation from friends, extended family, and other social support networks, with the potential to cause a feeling of isolation and the development of psychosocial problems [1, 9].

Exposure to stress and reduced social interaction during the pandemic significantly impacted adolescents’ mental health, leading to high rates of anxiety, depressive symptoms, and posttraumatic stress [10, 11]. These issues were shown to persist in the medium and long term due to the vulnerability of adolescence [2]. Although the negative impact decreased as social contact resumed, many adolescents continued to face mental health problems, particularly those related to chronic stress from pandemic confinement [12, 13].

During the pandemic, social distancing measures increased interaction between adolescents and their families at home, affecting intrafamily relationships and well‐being [4]. The pandemic created stressful conditions, such as economic challenges, remote education, marital conflicts, and intrafamily violence, all of which negatively affected family members’ mental health [4, 14, 15]. Conversely, it also provided a unique opportunity for family reconnection, memory‐making, and reevaluation of family priorities [16]. Affected by the discomfort resulting from losses, adversities, and weakening of vital bonds, families discover resilience from a challenging perspective [14]. Previous vulnerabilities in the family environment increased the fragility of social relationships and interactions [6, 17, 18].

The family plays a crucial role in the socialization of children and adolescents, especially during the pandemic. COVID‐19 has increased family time, highlighting challenges and opportunities for promoting adolescent mental health. The family environment and bonds significantly influence how adolescents navigate this period of insecurity and deprivation. Identifying positive family interactions can help adolescents cope with helplessness, failure, and despair, enhancing their potential for personal maturity and adaptation during the pandemic [5, 18].

Despite the existing international literature addressing the impacts of the COVID‐19 pandemic on adolescent development and mental health, important gaps remain. Most studies have been conducted in high‐income countries, with limited attention to the realities of the Global South, such as Brazil, where structural vulnerabilities, social inequalities, and fragile support networks strongly shape family dynamics and adolescents’ lives [1, 4, 5]. Moreover, quantitative approaches prevail [6], often overlooking the symbolic meanings that adolescents attribute to their lived experiences at home during social isolation. Few investigations have employed robust theoretical frameworks, such as symbolic interactionism, to examine how adolescents negotiate roles, redefine relationships, and construct new meanings within their families during crises. Addressing these aspects can bridge nursing and public health perspectives, thereby providing valuable evidence to inform family‐centered care and postpandemic health interventions. Therefore, this study aimed to analyze the experiences of family interaction and the social dynamics of adolescents in the context of social distancing during the COVID‐19 pandemic.

## 2. Methods

### 2.1. Study Type

This is an exploratory study with a qualitative approach [19]. This study aims to understand a phenomenon in a context, seeking new insights through a new light [19]. The study follows the Consolidated Criteria for Reporting Qualitative Research (COREQ) guidelines [20]. The qualitative approach was guided by symbolic interactionism and supported by inductive thematic analysis [21, 22], allowing for a deeper understanding of the meanings adolescents assigned to their experiences.

Understanding these relational and emotional dynamics is essential for nursing practice. Nurses, especially in primary healthcare, are on the front lines of detecting emotional distress, guiding family‐centered interventions, and implementing psychosocial support strategies. Strengthening this knowledge base contributes to the development of skilled and context‐sensitive nursing care in postpandemic settings, with the goal of improving adolescent well‐being and family resilience. In this regard, this study was guided by the theoretical lens of symbolic interactionism. This framework offers a distinct approach to the study of life and human actions, positing that the analysis of human behavior should be grounded in the concept of social acts. An individual actor perceives the actions of others and constructs a response based on the perceived intentions of those actions. Such intentions become symbolic, acquiring meaning ascribed by individuals rather than being inherently defined by the object itself [23, 24].

### 2.2. Study Location and Participants

The study was carried out in two state public schools in a small municipality located in (blinded for review). Inclusion criteria were as follows: (i) being between 10 and 19 years of age; (ii) being enrolled in one of the selected schools and having experienced remote learning during the COVID‐19 pandemic; (iii) having access to the internet; (iv) being enrolled from the 9th year of elementary school to the 3rd year of high school; and (v) providing informed assent and consent. Exclusion criteria included adolescents who were unable to communicate adequately, who did not complete the consent process, or who withdrew from the study before or during the interview.

Participants were selected using a convenience sampling strategy, based on their interest and availability to participate in the study.

### 2.3. Data Collection

Initially, the second author (X), a nursing undergraduate student trained by an experienced researcher in qualitative studies (X), contacted the school principal to present the research and outline strategies for its operationalization during the COVID‐19 pandemic. Based on joint decisions, it was decided to include one of the authors (X) in the school’s WhatsApp groups.

Subsequently, invitations were sent to teenagers to participate in the study in the schools’ WhatsApp groups with the research information [9]. The same researcher contacted students who expressed interest in participating. The students received information about the study, and a time was scheduled for guidance and receipt of the Informed Consent and Assent Form. Subsequently, with the consent of parents/legal guardians and the permission of the adolescents, a new time was scheduled to collect the signed forms. All biosafety measures to prevent COVID‐19 were taken in this process, including using masks, hand hygiene with alcohol gel, and interpersonal distancing [25].

The interviews were scheduled on a day and time convenient for the adolescents. The individual interviews were audio and video recorded remotely using the resources of the Google Meet platform [9]. The interviews were carried out from October to December 2020 by the second author (X), supervised by two nurses with Ph.D. degrees (X) and (X), with extensive experience in children and adolescent health, family interactions, and chronic diseases. One‐time interviews were conducted with each participant, with an average of 40 min, varying from 16 to 80 min. The interviewer had prior contact with the study population through online lectures about promoting mental health for adolescents in public schools during the COVID‐19 pandemic [9].

A semistructured script composed of two parts was used: The first referred to the sociodemographic characterization of the participants, such as age, sex, education, and housing conditions; the second part consisted of open questions related to the adolescents’ experiences at home with their family during the period of social isolation, with the following guiding questions: How was this period of social isolation due to the Coronavirus pandemic for you? How did you feel staying at home? What were the main difficulties you encountered staying at home during this time? [9].

The same guided interview questions were used with all participants, but the language was adapted to ensure age‐appropriate communication. The use of a young undergraduate interviewer facilitated rapport and identification, especially among younger adolescents, enhancing the quality and authenticity of responses.

The interviews were conducted until meaning saturation was reached, that is, when no new conceptual insights emerged and existing themes were explored with sufficient depth and nuance [26].

### 2.4. Data Analysis and Processing

After data collection, the interviews were transcribed verbatim. The participants’ nonverbal and verbal gestures and expressions were maintained [9]. To guarantee anonymity, the interviewees’ statements were identified by the letter A (adolescent), followed by cardinal numbers assigned to the participants (from A1 to A22).

The data were subjected to inductive thematic analysis [21, 22], guided by symbolic interactionism, and based on six stages: (1) familiarization with the data; (2) generation of initial codes; (3) search for themes; (4) review of themes; (5) definition and naming of themes; and (6) production of the report.

Quality criteria were ensured through multiple strategies, including adherence to COREQ guidelines, researcher training and supervision, data triangulation between team members during analysis, and rigorous documentation of all coding and theme development processes.

### 2.5. Ethical Aspects

The study strictly followed the ethical precepts proposed by Resolution No. 466 of December 12, 2012, of the National Health Council for research carried out with human beings. The (blinded for review) Institutional Review Board of the (blinded for review) approved the study, number (blinded for review).

## 3. Results

### 3.1. Characterization of Participants

Twenty‐two adolescents aged 14 to 18 participated in the study, with an average age of 16.18 (±1.18), primarily female (*n* = 17; 77.3%). Most self‐identified as White (40.9%) or mixed race (36.4%), followed by Black (18.2%) and Asian (4.5%). Regarding religion, Catholicism accounted for 45.5% of participants, Evangelical for 50.0%, and Jehovah’s Witness for 4.5%. Most participants lived in their own home (77.3%), while others lived in rented accommodation (13.6%). These characteristics are summarized in Table 1 and provide an essential context for understanding issues related to privacy, family composition, and the dynamics of sexuality‐related conversations within the household.

**Table 1:** Sociodemographic characteristics of participants (*n* = 22).

Variable	*N*	%	Mean ± SD
Gender			
Female	17	77.3	
Male	5	22.7	
Age (years)			16.18 ± 1.18
Race/ethnicity			
White	9	40.9	
Mixed race	8	36.4	
Black	4	18.2	
Asian	1	4.5	
Religion			
Catholic	10	45.5	
Evangelical	11	50.0	
Jehovah’s Witness	1	4.5	
Type of house			
Own	17	77.3	
Rented	5	22.7	
Household size			4.09 ± 0.92
Number of rooms			6.50 ± 1.68
Number of bathrooms			1.59 ± 0.59

*Note:* Values are presented as frequency (percentage) or mean ± (SD) standard deviation.

After analyzing the data, four themes were identified, as shown in Table 2.

**Table 2:** Themes developed through data analysis.

Theme number	Title
I	Caught by surprise: break with the world and the (re)discovery of home
II	Transformations in family interactions: conflicts and tensions versus reconciliation and resignification
III	Opportunities to reflect and be grateful: between fears, concerns, and gratitude

### 3.2. Themes Developed Through Data Analysis

#### 3.2.1. Caught by Surprise: Break With the World and the (Re)discovery of Home

The pandemic promoted a rupture with the previously known daily world, removing adolescents from their school environment and social activities. In addition, there was the imposition of changes in habits, behaviors, and interactions, especially regarding the impediment of interpersonal physical contact, such as gestures and attitudes of showing affection through hugs and kisses.

I was upset about not being able to go out, not being able to go on trips, and not having contact with people. I′m very affectionate, so I like kissing, hugging, and touching. This upset me in a certain way because when I went to school, I hugged my friends, talked, kissed them on the cheek, hugged the teachers, and always showed affection. A21, Female, 16 years old

Not being able to do the things I liked to do. Like, I went out with my friends to play ball, play basketball. Get something to eat, and now you can′t do that anymore, right? All public places are closed, and there’s nowhere to go. A20, Male, 16 years old

This process significantly influences adolescent development, which unfolds through mechanisms of identification and social interaction. Among the notable events in the changes in life dynamics, the adolescent’s longer time spent in the home environment stands out. There was closer contact and coexistence, with more interactions with family members.

I believe it was a period of many changes, we were kind of surprised, especially for me, as I′m in my third year (high school); I had all the expectations for the third year, and now we at home don′t have that same reality, so it was tough to get used to it. A7, Female, 17 years old

The meaning that adolescents attributed to social distancing varied based on their unique experiences, interactions, and perceptions, leading to different impacts. A prior habit of spending more time at home aided the adaptation process. Adolescents with less previous social interaction did not miss social contact during the pandemic. Moreover, their perceptions of well‐being at home influenced their adaptation.

Ah, I was fine (…) I′m not a person who goes out often, not because I don′t have friends, it′s just that I like staying home. A3, Male, 16 years old

Given that I didn′t go out much anymore, it didn′t get me down too much, but I went out very little to see my friends and I don′t go out. I miss hanging out with them in the square and playing in the street, but I didn′t go out much anymore, so it doesn′t make much difference to me. A6, Female, 17 years old

However, for some adolescents, the meaning of the mandatory measure of staying at home presented a conflicting nature and violated the adolescent’s dimensions of freedom, autonomy, and choices. For some adolescents, the period of social distancing represented a dimension of prison and confinement, causing intense anguish, especially among adolescents who had previous interactions and were away from home.

We are anguished about staying at home, about not being able to go out. We complain about school, but without it, it′s also horrible. A12, Male, 16 years old

I was very upset because it was tough; it was like having a life that before was almost all day away from home, and then the next day, I couldn’t even go out to the store; it was tough. A8, Female, 15 years old

#### 3.2.2. Transformations in Family Interactions: Conflicts and Tensions Versus Reconciliation and Resignification

The prolonged period at home represented an unexpected factor for the adolescent, who initially understood that this measure would be for a short period. During this period, dissatisfaction was caused by several aspects, including economic problems, conflicts in relationships and family interactions, arguments, establishing a new routine, and new assignments for the adolescent.

I couldn′t do anything that made me feel good; I was worried about economic problems, and my relationship with my family became difficult. I didn′t even care about the virus. A15, Female, 17 years

At first, I didn′t think it was going to be much, that we were going to stay at home for so long, and then I think that towards the middle, it was more difficult because there was more bad news, that sort of thing, and over time I just I got used to it and adapted to the new routine at home. A22, Female, 16 years.

The long period in the home environment imposed significant changes in the adolescent’s activities and routine, as there was a need to contribute to domestic chores and adapt to this new reality. Ambivalence was reported due to the intensification of coexistence at home and the gradual wear and tear over time.

The difficulties were that I had to do things I normally wouldn′t do if everything were normal, like housework. A1, Female, 17 years old

In the beginning, it was cool, like spending extra time with my family, and rest, too, but then it was a huge burden for me, you know? I had to help around the house. Not being able to go out either is horrible, you know, not having the security to even go to the market, because we get scared, but it’s like that. A13, Female, 18 years.

Sharing the home environment with family members for a prolonged period led, in some interactions, to the adolescent’s feeling of loss of space, freedom, and individuality.

My house, you know, I live with a lot of people; I have my parents, and I also have three brothers, so sometimes it felt a little oppressive, I don′t know, because there were a lot of people at home, but most of the time it was quiet. A22, Female, 16 years.

Furthermore, in this context, conflicting situations between adolescents were evident in family relationships, and these may have existed in interactions with family members and intensified with the long period of domestic coexistence. Difficulties in relationships between family members and the ensuing arguments were highlighted during social distancing and represented stressful factors. The complexity of the home situation required more significant effort from the adolescent to try to coexist harmoniously, avoid conflicts, and cope with these situations.

The biggest difficulty was my bad relationship with my father and brothers. A17, Female, 16 years.

Ah, it was a little irritating. Irritating because my family and I can′t spend much time together as there′s a bit of a fight. A13, Female, 18 years.

It was observed that, in particular families, the teenager remained alone at home during the pandemic due to the work activities of adults. Also, the difficulty and lack of dialogue between members of the family group were evident, contributing to the adolescent’s feeling of isolation and loneliness in the home environment.

Some people live in the same house but seem like strangers because they only look at their cell phones. A21, Female, 16 years old.

I live with my mother, and she is always working a lot, sometimes we don′t have much time to talk. A5, Female, 17 years.

Intergenerational conflicts, when experienced, intensified the negative effects during the COVID‐19 pandemic at home:

My family, my great‐grandmother and great‐grandfather, are strict and very religious, everything I did and did wrong was the reason for them to talk to me for hours, comparing me with cousins who are much better than me and who do everything right. This was and is a weight for me to carry. A18, Female, 14 years old

The establishment of routines with the family and the staleness in interactions for the adolescent led to a feeling of boredom and monotony.

I think it was sickening because sometimes it′s not so good to spend so much time with them, and it′s not so healthy to spend so much time with them. So, every now and then, it wasn′t so good. A1, Female, 17 years old

We were close, but we got even closer. Sometimes, it′s a little annoying. Putting up with things, like, you know, being around all day, the routine and everything, but other than that, it′s good. It′s good to spend time with family. But sometimes, it′s a little annoying, but other than that, it′s good. A11, Female, 15 years old

However, certain families sought strategic coping actions and developed new group activities, promoting more significant interaction between members and breaking the monotonous routine during the pandemic.

I stayed at home; it was very boring at first because I had nothing to do, but I started doing physical exercise with my mother and my cousin, and then it was something that relaxed my mind more, and it was better for us. A10, Female, 14 years old

I managed to balance reading and doing these things… I even learned how to make bread during the pandemic (laughs). A8, Female, 15 years old

The pandemic provided opportunities for reflection and maturity for the adolescent, creating a moment of learning amid the adversities and afflictions experienced. Getting closer to God, resilience, and exercising patience were mentioned as changes noticed by some teenagers:

I managed to focus more on my things and work more on myself, on changing a lot. I changed during this period; I talked to myself and became slightly calmer. It was supposed to be the opposite, but I believe it was pretty peaceful for me. A5, Female, 17 years

So, it was also a moment for me to get closer to God, to ask for more for people′s lives; I think it was also a moment, how can I say, a beautiful moment in my life because I saw many people become closer, I saw many people getting more involved, so I was grateful, I felt relieved. A21, Female, 16 years old

Between conflicting situations and family tensions, the need for dialogue and attempts at reconciliation emerged in the search to alleviate everyday problems and improve coexistence among members of the family.

Look, from one perspective, it was excellent, but it was also awful because we have extreme opinions, everyone at home does, and so it was (…) there was a lot (…) a lot of bullshit like that. But there’s nothing like a good conversation. It′s a curse. But what are you going to do (laughs)? A19, Male, 17 years old

We had some problems (in the family), so I tried to offer my greatest support, my greatest support so that things didn′t get worse than they already were. So, I′ve avoided arguments and fights to make the atmosphere a little lighter and to alleviate the situation. A10, Female, 14 years

In this process, some adolescents reported the experience of strengthening ties with other members of their family in a process of maturation, reflection, and negotiations in their family life during the pandemic period. Due to the greater distance between family members before the pandemic, the period of social distancing allowed for greater possibilities for interactions, reframing the importance of family and establishing dialogue and connections between members.

My God! (…) I had to be patient because we didn′t used to spend as much time together. I had to learn to be more patient because people are different from me, but it was an experience that I think was good because now I′m a different person. A8, Female, 15 years old

I believe that many families were restored at home by this time. I never had a good relationship with my father, (…) he never understood me, and we were never very close (…). I don′t know if he also felt the same as me, that people could go at any moment, that this virus is there to get both poor and rich, both black and white, it′s a virus that came to get everyone, it doesn′t choose who catches it (…) My relationship with him has improved a lot (…); we talk more and get along better. A21, Female, 16 years old

#### 3.2.3. Opportunities to Reflect and Be Grateful: Between Fears, Worries, and Gratitude

In the pandemic context, adolescents had to deal with the fear of contagion from family members, even distant ones, friends, and people close to them, while facing death, loss, and grief.

My father lives in [another state], and he got infected with the virus, and I felt awful at that time, during that period. No one here in my house caught it, which was very comforting, but with my father, I was very worried because I didn′t see him much. But he was fine in the end; everything was fine. A6, Female, 17 years old

I have a lot of contact with my grandmother, right? She is old, old like that. So, I was a little scared. A12, Male, 16 years old

On the other hand, for some adolescents, the COVID‐19 pandemic caused less concern regarding the family group, the implementation of prevention measures being one of the reasons highlighted, as well as the feeling of not being affected by the disease.

Partially, about the virus, I didn′t worry too much (…) because I knew they were respecting the quarantine. But now, going out… because if you have to go out alone, it′s awful, and others are more worried than me, and it′s become difficult. A19, Male, 17 years old

I was scared, yes, but I also tried to think about other things, more positive things. A4, Female, 16 years old

The experience of these stressful situations led to moments of reflection and introspection by the adolescent, besides the search for the meaning of life.

Sometimes we complain so much. There are people worse than us, so it was a moment when I reflected a lot when I said Thank God that I have a roof over my head, that I have food, and that I′m healthy because many people are in a hospital bed or many people couldn′t even bury their loved ones because of this virus. I′m very grateful; it was a moment of reflection, gratitude, and concern for my family. I know what it′s like to lose someone dear, in that my beloved grandmother died of COVID, so it was something that had no explanation. A21, Female, 16 years old

## 4. Discussion

The study explored how adolescents interacted with their families during COVID‐19 social distancing. Staying at home brought significant changes to their daily lives, including school closures, separation from peers, limited extracurricular activities, reduced autonomy, and new responsibilities at home. Alongside these changes, adolescents faced fears about the pandemic, concerns for their family’s health and well‐being, and economic and social impacts. These challenges led to daily conflicts within family interactions but also prompted efforts at reconciliation, adopting coping strategies, and developing family resilience.

The pandemic posed particular challenges for adolescents, given that they were undergoing a critical stage of development and performing social roles in everyday interactions that are, to some extent, expected to align with socially constructed identities, as highlighted in Goffman’s work. The intensified coexistence within family settings required the development of distinct performances, especially those enacted on the front stage—that is, behaviors expected by family members. In contrast, backstage behaviors, which involve more authentic or experimental identities, became increasingly difficult to express [27]. Despite these challenges, the study reveals a healthy pursuit by adolescents of meaningful lived experiences—a dimension that must be considered in healthcare, particularly in Nursing, given its comprehensive perspective and legitimized presence within health services [28].

Living with restrictions and concerns related to COVID‐19 increased emotional stress and reduced life satisfaction among the participants. This dimension certainly contributed to the attribution of new meanings to the experience of adolescence, given the unexpected emergence of symbolic references [23]. The high levels of stress experienced during the pandemic, resulting from hardship due to the loss of financial resources, unemployment [16, 17], concern and fear of disease contagion, and the imminent death of loved ones [1], had an impact on disrupting families’ everyday interactions and routines [4]. Given the scenario experienced, many families had their mental and emotional resources exhausted [17].

From a symbolic interactionism perspective, it is understood that the meanings of family experiences reported by adolescents are understood not as given a priori, but as constructed through daily interactions with their peers and family members [23]. This process of role‐taking, the ability to put oneself in another’s place to guide one’s own behavior [29], emerged in the adolescents’ accounts, particularly when they described adopting protective attitudes toward their parents in response to fears related to COVID‐19.

Another stressful factor for our adolescents was restricting contact with peers and participating in extracurricular activities, such as sports, dancing, music classes, and social events. Important behavioral changes with significant influence on health were introduced during the pandemic, with greater use of computer screens, tablets, and cell phones, impaired sleep patterns, and physical inactivity [1, 17]. From the adolescent’s perspective, contact with friends and social connections were their most significant concerns when compared to other problems aroused by the pandemic [1], given the symbolic meaning attributed [30] to interactions with peers, in line with the findings of this study. These results highlight the need for ongoing health monitoring of adolescents, even after the pandemic, because these experiences significantly affect their development, especially as peer identification is crucial in shaping their identity.

Extended online engagement has reshaped how adolescents relate to themselves and others, a process that can be interpreted through Goffman’s dramaturgical theory. In digital contexts, social media and interactive platforms operate as stages for identity performance, where individuals strategically manage impressions. Studies on bloggers and *Second Life* users highlight practices such as persona embellishment, anonymity, and disconnection between real and virtual selves [31]. These dynamics help explain why, during social isolation, online interaction assumed a central role for adolescents, functioning both as a means of belonging and as a space for identity reaffirmation amid emotional and familial constraints.

The intensification of online life during the pandemic can also be interpreted through Guy Debord’s critique of the “society of the spectacle,” in which social relations are mediated by images and appearances prevail over being [32]. Within this framework, social media function as showcases of carefully curated performances, where adolescents often feel compelled to sustain an idealized self‐image. This spectacular logic of self‐exposure and constant comparison reinforces Goffman’s [27] notion of *front-stage* dynamics, but with greater intensity, given the relentless scrutiny of digital audiences. Such performative overload may produce significant psychological consequences, including anxiety, feelings of inadequacy, and depression, particularly among youth undergoing identity formation. Empirical evidence indicates that excessive social media use is associated with depressive symptoms and diminished self‐esteem, especially when personal validation becomes dependent on likes and comments [33]. Thus, the hyper‐connected life of the pandemic period emerged as both a refuge and a site of emotional vulnerability.

The meaning of staying at home experienced by adolescents emerged from their interactions and interpretations of the situation, from which they acquired different behaviors according to the conditions they faced [30]. A long time at home with the family is essential to the adolescent’s mental health [5]. Our findings showed that previous family interactions and the meanings attributed to home directly influenced adolescents’ experiences and their adaptation process during the pandemic. In other words, the symbolic meanings previously associated with the family were key determinants of more positive experiences during the pandemic period.

The imposition of full‐time coexistence with the family nucleus created the need to negotiate the sharing of home spaces, which influenced the restriction of the adolescent’s privacy. Privacy is crucial for the subject’s unique identity, which is under construction during adolescence [17, 34]. The constitution of the family structure must be considered, such as single parents or large families living in confinement in small environments [16], which directly interferes with family interactions. Developing personal identity and discovering the social space to which the subject belongs are dynamics foreshadowed during adolescence and require the separation of parents or legal guardians. The home configuration may have deficient space and a lack of privacy, allowing episodes of aggression and conflicts because of this intensified coexistence [34].

The present study showed antagonistic responses to the pandemic context, with some adolescents and families able to adapt better and promote better interactions than others. Parents who did not struggle with mental health were able to efficiently help their children face stressful factors related to the pandemic [4]. They could establish constructive communication and strategies for conflict resolution [16]. In these families, parents were less likely to transfer negative feelings to other members, contributing to maintaining health and well‐being during the pandemic [4]. It can also be assumed that, within these families, there was greater freedom and autonomy for adolescents to explore backstage performances more effectively [27].

An Australian study showed that some families found opportunities for growth, development of tolerance, patience, understanding, and coping strategies [16], which is in line with the findings of this study. Among the factors that influenced the formation of a positive context were highlighted internet access, a safe home space, sufficient food, more time for interaction with the family, a support network, good health, and financial stability [16].

However, many families have experienced deteriorating mental health among their members, exhibiting symptoms such as depression, anxiety, and posttraumatic stress [16]. The pandemic exacerbated existing mental health issues and intensified vulnerabilities in certain families [16, 35]. Social restrictions and the economic impact of the pandemic further contributed to these challenges [35], along with the profound loss of loved ones, all negatively affecting mental health [35]. In this context, adolescents were significantly impacted, as reflected in this study, where concerns related to family income and relationships often outweighed fears about the disease itself.

The study identified tensions in interactions between adolescents and their families, including intergenerational conflicts. Consequently, adolescents often feel lonely due to limited interaction with other family members. Adverse family dynamics and lack of social support were strongly linked to mental health issues among adolescents [12], with high levels of loneliness exacerbating these challenges [7]. Adolescents experiencing conflicts with their parents during the COVID‐19 pandemic reported more severe depressive symptoms and lower life satisfaction compared to those in less conflict‐ridden environments [1].

When examining the emotional impacts of significant crises like Hurricane Katrina in the USA and the tsunami in Sri Lanka, studies emphasized the critical role of positive family relationships in supporting adolescents facing symptoms of depression, and posttraumatic stress [36, 37]. Therefore, interventions and supportive measures for families, particularly those in vulnerable conditions, are crucial to protect and foster emotional adjustment within the family system during crises such as the COVID‐19 pandemic [35].

During the pandemic, nursing care for adolescents should focus on building a healthy support network and involving families in care plans to manage stress. The study’s strength lies in the significant impact of family interactions on adolescents’ well‐being during COVID‐19 social distancing and the need for postpandemic interventions to support their health and well‐being. However, we acknowledge some limitations, such as focusing solely on adolescents and not assessing previous mental health issues among participants and their families. New studies focusing on postpandemic family interactions should be conducted to identify the strategies and needs of this population and the impacts on the adolescents’ development.

In addition to the dimensions already discussed, it is important to consider the gender profile of the participants, given its potential influence on the narratives produced in the study. Most participants in this research were female, which may have shaped the findings, since family experiences, as well as the ways emotions and conflicts are expressed, are often shaped by gendered social constructions. Gender is a fundamental analytical category for understanding how power relations structure social experiences [38]. In this sense, the predominance of female participants may have limited the emergence of narratives related to masculinities and the particular ways in which adolescent boys experience family life. This imbalance reinforces the need for caution when generalizing the results and highlights the importance of future research that includes a broader range of gender expressions.

## 5. Conclusion

The meaning of the COVID‐19 pandemic was understood from adolescents’ interactions with their families in the home environment, in which adolescents adopted different roles to face the demands and challenges presented by social distancing and other safety measures. Concern about the disease, fear of death, financial problems, loss of adolescent autonomy and space, and intrafamily conflicts were present in the reports. Confronting the situation required attempts at reconciliation and negotiations for a better coexistence in the face of the stressful situation. The COVID‐19 pandemic also influenced the maturation process, with reflections and improved resilience capacity for some adolescents, along with recognizing the value of family in the face of adversity.

Based on these findings, we recommend that nursing practice, school‐based mental health programs, and public policy initiatives consider the lived experiences of adolescents during the pandemic. It is essential that this historical context be acknowledged and incorporated into adolescent health assessments and nursing consultations. Moreover, the use of tools that allow for the exploration of family dynamics should be encouraged, as they offer valuable insights into the adolescent’s support networks and potential stressors. Such approaches can contribute to more comprehensive and context‐sensitive care.

### 5.1. Key Messages

1. Longitudinal health monitoring for adolescents in postpandemic times is necessary due to the profound impacts on their development.
2. Despite the negative impacts on family interactions across various dimensions, resilient coping strategies were possible.
3. Family interactions and dynamics of adolescents need to be better investigated and uniquely addressed in postpandemic times, and the perspectives of all family members must be considered.

## Ethics Statement

The study strictly followed the ethical precepts proposed by Resolution No. 466 of December 12, 2012, of the National Health Council for research carried out with human beings. The Ribeirão Preto School of Nursing Research Ethics Committee of the University of São Paulo approved the study, number 4.354.107/2020.

## Consent

All participants gave written informed consent for participating in the study.

## Conflicts of Interest

The authors declare no conflicts of interest.

## Author Contributions

Nayara Gonçalves Barbosa, Thaianne Cristine Gadagnoto, and Lise Maria Carvalho Mendes conceptualized the paper, and Lucila Castanheira Nascimento was involved in funding acquisition. Nayara Gonçalves Barbosa, Lise Maria Carvalho Mendes, and Flávia Azevedo Gomes‐Sponholz developed the methodology. Thaianne Cristine Gadagnoto collected the data, and Tatiane Geralda André, Giovanna Cristina Machado Kayzuka, Diene Monique Carlos, Nayara Gonçalves Barbosa, Thaianne Cristine Gadagnoto, Lise Maria Carvalho Mendes, Flávia Azevedo Gomes‐Sponholz, and Lucila Castanheira Nascimento analyzed the data. The project administration was undertaken by Nayara Gonçalves Barbosa, under the supervision of Flávia Azevedo Gomes‐Sponholz and Lise Maria Carvalho Mendes. Nayara Gonçalves Barbosa, Lucila Castanheira Nascimento Lise Maria Carvalho Mendes, Thaianne Cristine Gadagnoto, Giovanna Cristina Machado Kayzuka, Tatiane Geralda André, Diene Monique Carlos, and Flávia Azevedo Gomes‐Sponholz drafted the original manuscript, and all authors contributed to reviewing and editing it.

## Funding

Research reported in this publication was supported by the National Council for Scientific and Technological Development (CNPq), Brazil, Process number 309528/2021‐6.

## Data Availability

The data that support the findings of this study are available upon request from the corresponding author. The data are not publicly available due to privacy or ethical restrictions.
